# Grammatical class modulates the (left) inferior frontal gyrus within 100 milliseconds when syntactic context is predictive

**DOI:** 10.1038/s41598-019-41376-x

**Published:** 2019-03-18

**Authors:** Kristof Strijkers, Valerie Chanoine, Dashiel Munding, Anne-Sophie Dubarry, Agnès Trébuchon, Jean-Michel Badier, F.-Xavier Alario

**Affiliations:** 1 Aix Marseille Université, CNRS, LPL, 13100 Aix-en-Provence, France; 2 Aix Marseille Université, Institute of Language, Communication and the Brain, Brain and Language Research Institute, 13100 Aix-en-Provence, France; 3 Aix Marseille Université, CNRS, LPC, 13331 Marseille cedex 3, France; 4 Aix Marseille Université, INSERM, INS, 13005 Marseille, France

## Abstract

The current study set out to examine the spatiotemporal dynamics of predictive processing during syntactic processing. To do so, we conducted an MEG experiment in which we contrasted MRI-constrained sources elicited by nouns and verbs when they were preceded by a predictive syntactic context (i.e., possessive pronouns for nouns, and personal pronouns for verbs) versus a non-predictive syntactic context (visually matched symbols). The results showed rapid (from ~80 ms onwards) noun-verb differences in the left and (to a lesser extent) right inferior frontal gyri (IFG), but only when those nouns and verbs were preceded by the syntactically predictive context (i.e. their corresponding pronoun). Furthermore, the contrast between possessive and personal pronouns that preceded the rapid noun-verb modulations in the (L)IFG also produced differences in source activation in various regions of the prefrontal cortex (the superior frontal and orbitofrontal cortex). We suggest the data show that syntactic unification manifests very early on during processing in the LIFG. The speed of such syntactic unification operations is hypothesized to be driven by predictive top-down activations stemming from a domain-general network in the prefrontal cortex.

## Introduction

Prediction has become a key construct to explain the speed and efficiency of brain processing^1–3^. In language research, prediction is taking a prominent role in empirical research and theory^4–6^, though the term prediction may mean different things to different researchers^7^. Here, we will use prediction to refer to the mechanism whereby the language system retrieves information prior to the processing of the bottom-up input, and where this pre-activated information is not the mere consequence of activation spreading forward associatively from the input (e.g., the input ‘cat’ may activate ‘dog’ merely because they share semantic features). Put differently, prediction will refer, here, to the pre-activation of linguistic knowledge driven by top-down signals rather than by bottom-up signals.

The literature counts on a wealth of evidence, stemming from various paradigms, indicating the existence of such top-down driven predictions in language processing (for reviews see)^1,7,8^. By far the most explored neurophysiological component that bears on this issue is the N400^9^. The more predictable an upcoming word becomes (e.g., ‘*palm trees*’ versus ‘*tulips*’ following the sentence ‘*To make the hotel look like a tropical resort, they planted a row of…*’), the easier lexico-semantic processing becomes, as indexed by an N400 reduction^10–12^. However, given that the N400 is a relatively late component, and that linguistic processing has been found to affect brain processing between 100 to 200 ms earlier than that^13,14^, it is not clear whether these results relate to word anticipation or facilitated post-lexical/semantic integration^13,15,16^. Stronger evidence comes from an elegant paradigm where noun predictability modulated N400 amplitude already on the article preceding the noun (e.g., ‘*a*’ vs. ‘*an*’ following the sentence ‘*The boy went outside to fly…*’, thus anticipating “kite” or “airplane”, respectively, with an N400 reduction for the former)^17,18^. However, recent failures to replicate this effect cast some doubt on its generalizability^19^ (see also)^20^. Furthermore, the available evidence does not reveal the mechanism by which predictive anticipation affects the spatiotemporal dynamics of linguistic processing.

To gain insights on the role of prediction in language processing and the spatiotemporal dynamics that may underpin it, we here opted to follow a different approach and rationale than those described above. While most cognitive neuroscience studies on language prediction focused on predictive contexts (semantic, phonological or grammatical) that constrain processing at the word-level, and measured prediction as an increase of the likelihood that a specific word will appear, we here set out to explore the predictive coding of language through the functional lens of syntactic unification: that is, the process of binding different memory representations (morphosyntax) into larger syntactic structures (e.g. possessive pronoun + noun = noun-phrase structure)^21^. Concretely, we explored contexts that constrain to specific grammatical categories (for behavioral demonstrations see)^22–24^. To do so, our experimental design resorted to nouns and verbs, which can be preceded by different types of personal pronouns: possessive versus personal pronouns before nouns and verbs, respectively, but never the other way around^25^. The exclusive relationship between these specific closed- and open-class words affords unambiguous unification operations where the structural frame of a possessive pronoun can only be attached to a noun-phrase and that of a personal pronoun to a verb-phrase. Consequently, such determined structure provides an ideal environment for predictive processing^26–28^ and could allow investigating how the retrieval of noun and verb categories is affected by such predictive syntactic context. With this aim in mind, the reliance on noun and verb categories are particularly useful neural markers.

Many neuropsychological, behavioral and neuroscientific data show that the processing of nouns and verbs recruit, in part, different neural circuitries. Broadly speaking, the dissociation follows a posterior-anterior gradient, where nouns activate more strongly (left) temporal brain regions while verbs activate more strongly (left) fronto-central brain regions^29–31^ To be fair, the actual contrast between the neural processing of nouns and verbs is somewhat more complex, with some studies reporting evidence inconsistent with such posterior-anterior dissociation^32,33^. Also, the level of representation (i.e., grammar versus semantics) for which noun-verb contrasts become apparent remains an open issue^30,32^. Despite current debates on the nature(s) of the noun-verb distinction, which are beyond the scope of the current study, here we exploit the fact that nouns and verbs are frequently reported to elicit dissociations in fronto-temporal activation.

Importantly, this (partial) fronto-temporal dissociation between nouns and verbs has also been observed with magnetoencephalography (MEG), the technique also employed in the current study^34,35^. By focusing on pronouns that either constrain to upcoming nouns or verbs, one can take advantage of the posterior-anterior cortical dissociations observed between nouns and verbs as a marker to trace when and how their processing is modulated by syntactic predictability. To measure that syntactic predictability we will therefore contrast, by means of MEG, the time-course of cortical area activations elicited by nouns or verbs when preceded by their corresponding pronouns (i.e., predictive context, where nouns are preceded by possessive pronouns and verbs by personal pronouns) compared to when they were preceded by visually matched non-informative symbols (i.e., non-predictive context).

With this experimental set-up, which thus focusses on grammatical predictability (rather than word-specific probability), we can explore three different sets of predictions: (1) at the lexical level, which concerns the main objective of the current study, to investigate whether syntax promotes predictive grammatical class activation (nouns vs. verbs); (2) at the sentence level, which is a secondary and more exploratory objective, to investigate the spatiotemporal dynamics underlying syntactic unification per se; and finally, (3) at the pronoun level, which is an exploratory objective, to gain descriptive insights on the spatiotemporal dynamics elicited by pronoun knowledge. With respect to (1), if syntactic unification promotes predictive processing^26–28^, we predict an interaction between ‘predictability’ (where pronouns can indicate an upcoming noun or verb) and ‘grammatical class’ (noun vs. verb), where we should observe a different noun-verb effect in the neuromagnetic response in function of the presence versus absence of prior pronoun information. Specifically, we predict that the noun-verb contrast should elicit an earlier (and stronger) posterior-anterior dissociation in the predictive syntactic unification context compared to when no syntactic unification is possible. With respect to (2), and above and beyond the potential effect on the memory representations (i.e., the anterior-posterior brain dissociations between the lexical representations of verbs and nouns), brain regions sensitive to phrasal-level syntactic operations should be affected by the presence or absence of pronoun information. We therefore expect significant interactions in brain regions associated with syntactic processing such as the left inferior frontal gyrus (LIFG)^21,28,36,37^ or (anterior) superior temporal cortex^36,38–41^, when the presence of pronoun information can predict the upcoming phrase-structure (i.e. when syntactic unification is possible) compared to when no meaningful pronoun information is available to the system (i.e. when no syntactic unification is possible). The objective here, thanks to use of MEG, is to explore the time-course of when those putative brain regions associated with the binding of syntactic information manifest. Finally, with respect to (3), by examining the brain regions activated prior to the presentation of the noun and verbs, that is, during pronoun processing, we may gain tentative insights on when and how pronoun information starts affecting the spatiotemporal dynamics of subsequent morphosyntactic (1) and/or syntactic unification (2) processing.

## Results

Participants engaged in a lexical decision task on the presentation of the second stimulus, where they had to push a response button if presented with a nonword (instead of a noun or verb; for more details see Methods section). Behavioral performance on the task was very high. On test trials (i.e. pseudo-words; around 11% of the trials), there were 93.3% of hits (SD = 12.7), 6.7% of misses (SD = 12.7). There were no false alarms in non-target trials (SD = 0).

**Figure 1:**
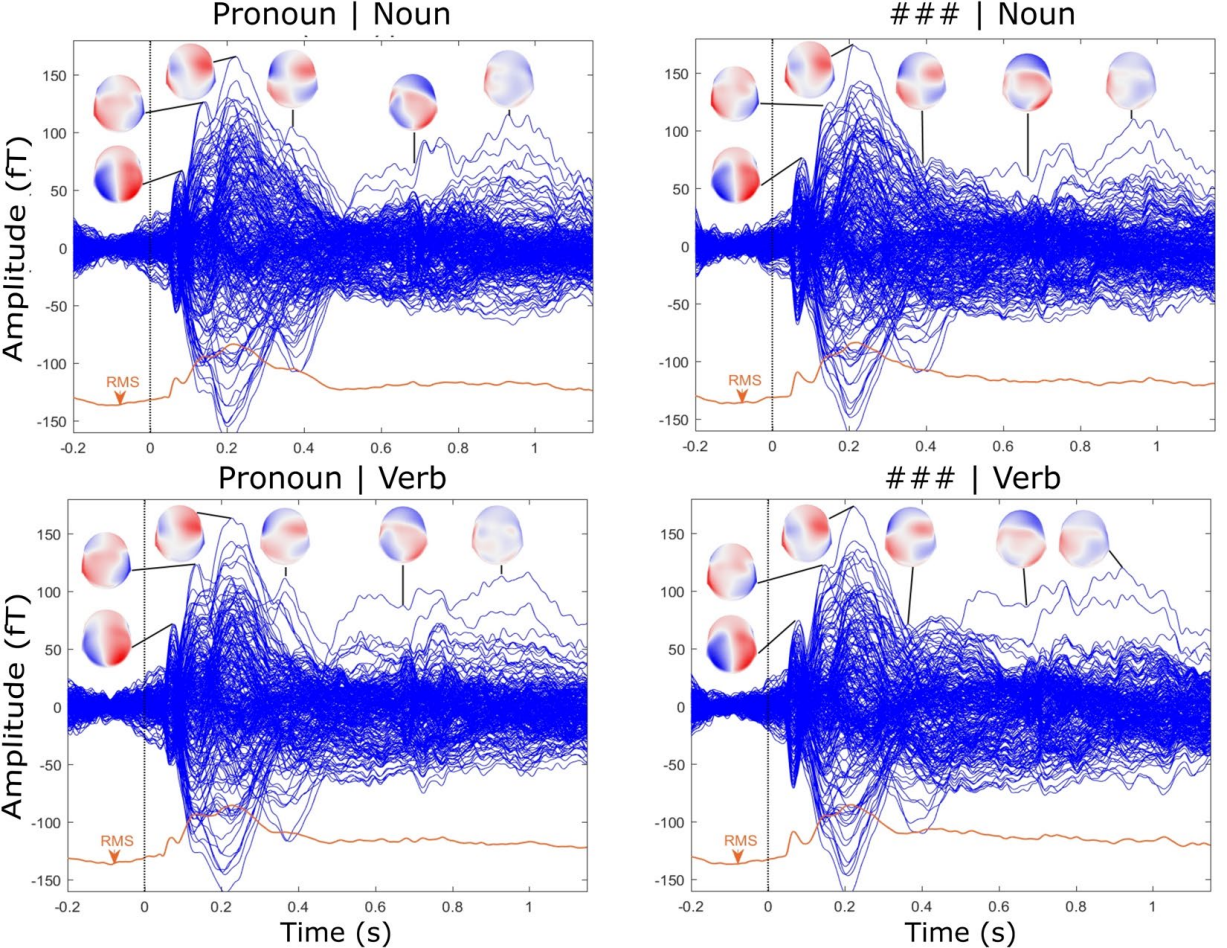
Event-related field potentials (ERFs) of all channels and their RMS per condition with topographic maps at peak latencies. The upper left panel represents the possessive pronoun + noun condition, the upper right panel the hashmarks + noun condition, the lower left panel the personal pronoun + verb condition and the lower right panel the hashmarks + verb condition. For all panels, the X-axis displays time in seconds (s) from pronoun/hashmark onset and the Y-axis the amplitude of the ERF in femtotesla (fT). Blue lines represent the individual channels and red lined the root mean square (RMS) over all channels. On the topographic maps blue corresponds to negative going amplitudes and red to positive going amplitudes.

With regard to the neuromagnetic data, Fig. 1 provides an overview of the responses in sensor-space (and the RMS over all channels) per condition and their topographic distribution at peak activation. Given that our study has no relevant predictions concerning sensor-space, but was entirely designed to assess the time course of prediction-specific cortical source activation, we will dedicate no further attention to it. For the source-space data, we ran mass univariate analyses on the source time-series (every 25 ms from stimulus onset until 1200 ms post-stimulus onset) for each ROI (i.e., whole-head analyses) (for more details please consult the Methods section). Given that the focus of our study concerns the interaction between Syntactic Predictability (‘predicted’ vs. ‘unpredicted’) and Grammatical Class (‘noun’ vs. ‘verb’) **(see Introduction)**, we describe these interactions first. For completeness, we also report the main effects of Predictability and Grammatical Class in order to demonstrate that they do not confound the data observed for the interactions. For all results, only significant FDR-corrected effects are reported; all non-reported contrasts (e.g., in regions not mentioned) were not significant.

### Interaction between syntactic predictability and grammatical class

Source activation patterns sensitive to the interaction between Syntactic Predictability (pronouns vs. ##) and Grammatical Class (noun vs. verb) were twofold: (1) In the left and (to a lesser extent) right inferior frontal gyri (IFG), we observed a significant interaction during both the pronoun interval (0–600 ms) and the noun/verb interval (600–1200 ms); (2) In the prefrontal cortex, bilaterally, we observed a significant interaction during the pronoun interval only.

**Figure 2:**
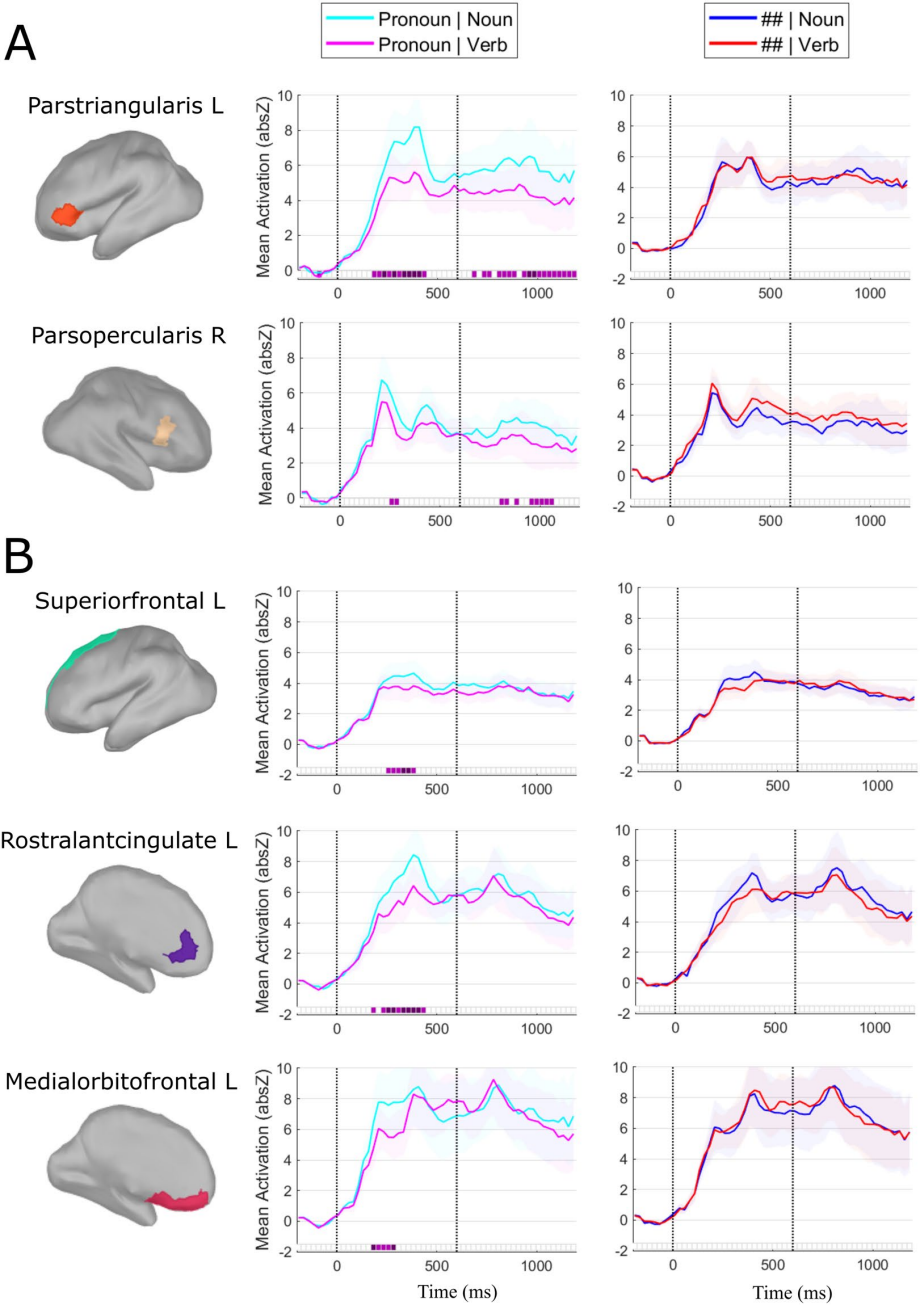
ROIs and time-course of the significant interaction effects between Syntactic Predictability and Grammatical Class. (**A**) Significant interaction contrast in bilateral IFG during the pronoun- (0–600 ms) and noun/verb-interval (600–1200 ms; lateral-view). (**B**) Significant interaction contrast in left prefrontal cortex during the pronoun-interval (medial-view) (note that the effect was significant bilaterally, but for presentation purposes we have only plotted the left prefrontal regions). Left: The ROIs showing significant differences are highlighted in color onto a morphed cortical reconstruction of a template brain. Right: The mean source activity (Y-axis) over time (X-axis) is plotted for each of the ROIs. The first 200 ms (−200 ms to 0 ms) correspond to the baseline, the next 600 ms correspond to the function word interval, and the last 600 ms (starting from the dotted vertical line) correspond to the content word interval. Purple squares on the X-axis (25 ms time steps) correspond to FDR-corrected p-values (light purple p < 0.05, dark purple p < 0.01) of significant interaction between Syntactic Predictability (pronoun vs. ##) and Grammatical category (noun vs. verb contrast). The corresponding pairwise comparisons within each predictability condition were also significant.

With respect to the first pattern, the interaction consisted in grammatical class effects being present for the predictable (pronoun) condition but not for the unpredictable (##) condition. When comparing nouns *vs*. verbs (600–1200 ms interval), we observed enhanced source activity in the left pars triangularis (and weaker, but significant, in the right pars opercularis) for the nouns compared to the verbs, starting as quickly as 80 ms after their onset and continuing throughout (almost) the entire interval **(**Fig. 2A**)**. We observed the same significant effect in the right pars opercularis, though clearly weaker and starting later (from 175 ms after noun/verb onset) compared to the effect in the left pars triangularis **(**Fig. 2A**)**. Furthermore, a similar effect was found for the pronouns (0–600 ms interval), with enhanced source activation for the possessive compared to the personal pronouns in the left pars triangularis between 200 and 400 ms after their onset (and again a weaker and shorter, though significant, effect in the right pars opercularis) **(**Fig. 2A**)**. Importantly, the Non-predictive condition showed no significant differences, neither in the pronoun nor in the noun/verb intervals **(**Fig. 2A**)**. In sum, in (left) IFG we observed an early difference in cortical activation between nouns and verbs when preceded by their corresponding pronoun context, which was absent when preceded by meaningless symbols. Note further that, contrary to our prediction, no typical anterior-posterior dissociation was found between verbs and nouns, neither in the Predictable nor in the Unpredictable conditions. In fact, the observed noun-verb differences in the Predictable context showed the reverse pattern to what is typically observed, with enhanced activity in LIFG for the nouns, not the verbs.

With respect to the second pattern, we observed in the superior frontal, rostral anterior cingulate and medial orbito-frontal cortex bilaterally (for which we use the group-label pre-frontal cortex; ‘PFC’) a significant interaction between ‘Predictability’ and ‘Grammatical Class’ in the pronoun-interval only: Between 200 and 400 ms after stimulus onset, the possessive pronouns elicited more enhanced source activity than the personal pronouns **(**Fig. 2B**)**. As above, the Non-predictive condition showed no significant differences, neither for the hash-marks presentation nor for noun/verb presentation **(**Fig. 2B**)**. In sum, we again find a significant interaction between Syntactic Predictability and Grammatical Class; interestingly, this time it is in the prefrontal cortex (PFC) outside traditional language areas and only seen for the contrast between possessive and personal pronouns.

### Main effects

Main effects of Syntactic Predictability (i.e. pronouns vs. hash marks irrespective of grammatical class), displayed two distinct patterns. First, during the pronoun interval (0–600 ms), several regions in occipital and middle temporal cortex showed stronger source activations for the non-predictive compared to the predictive conditions, starting as early as within the first 50 ms in occipital cortex and 150 ms in temporal cortex. These possibly reflect visual and familiarity differences between hashmarks and meaningful pronouns. Second, within frontal cortex, pronouns elicited stronger source activations compared to hash marks, initiating 200 ms after stimulus onset, especially for motor and somatosensory cortices. The direction of these effects is opposite to that observed in occipital and temporal regions described above, and the effects extended into (and are strongest for) the noun/verb interval. These arguably reflect processes related to the task (nonword identification) and motor preparation. That said, we will not speculate further on what these main effects may signify, because the current scope given by our design is tailored to test the interaction effects. In this regard, more important is the observation that none of these main effects falls within the ROIs where we observed the above discussed interaction effects.

Finally, main effects of Grammatical Class were present in all ROIs where we also observed significant interactions with predictability (as described above), and were thus entirely carried by the predictive condition. Small main effects of Grammatical Class only (i.e. noun vs. verb irrespective of predictability), were observed in the right hippocampus and the right lateral orbitofrontal cortex. These started around 100 ms before noun/verb presentation, with verbs eliciting stronger source activations than nouns and extending up to 200 ms after noun/verb presentation. These regions and their time-course were not expected and do not afford a straightforward interpretation, beyond the fact, perhaps, that they are likely unrelated to grammatical class per se, and thus may indicate stimulus and/or task related noise in those ROIs. Rather, the important message here is that these effects are of a different nature (sign is reversed compared to the interactions) and fall outside the critical ROIs of the interaction contrast, making it unlikely they pose a confound or problem for the interpretation of the interaction effects. To conclude, as was the case for the interaction contrasts, we again did not find any significant anterior-posterior dissociations between verbs and nouns, contrary to what was predicted based on the available literature. Given that we have no interest in the main effects we do not include their figures here. However, those figures (and their statistics) are available in the online repository of our data (https://blricrex.hypotheses.org/le-crex/sharedprojects; see also Methods Section at the end of the manuscript).

### Baseline correction

Before starting the discussion, a technical aspect of our analyses must be discussed. We have analyzed the pronoun and noun/verb time-windows in a single analysis with a pre-stimulus baseline, rather than baseline-correcting the MEG responses to the pronouns and the nouns/verbs separately (see Fig. 2A and Methods section). One may wonder whether this is the correct procedure and whether the above reported early interaction effects in the (L)IFG aren’t merely the continued response to the prior pronoun information. Indeed (and not surprisingly), when analyzing the data with a baseline-correction 100 ms prior to the presentation of the nouns/verbs, the early interaction between ‘Syntactic Predictability’ and ‘Grammatical Class’ in the (L)IFG no longer reaches the significance threshold (see online repository). That said, given the objectives specified in the current study of wanting to investigate how syntactic context may affect subsequent processing, we believe that a baseline correction of the entire syntactic sequence (i.e., prior to the pronoun information) is the better approach. The goal of baseline correction is to reduce (hopefully remove) task- and manipulation-irrelevant noise prior to the presentation of critical items. In the current paradigm, the pronoun interval preceding the presentation of the nouns and verbs is not task-irrelevant. Thus, the fact that the early (L)IFG modulations seen on nouns vs. verbs are affected by the pronoun-modulations that precede them is precisely a critical observation. Therefore, the correct baseline definition for the current data should be before the task-related constituents, not in the midst of them; with an alternative baseline, the critical information targeted by the design might be lost (see for similar rationale)^42^.

**Figure 3:**
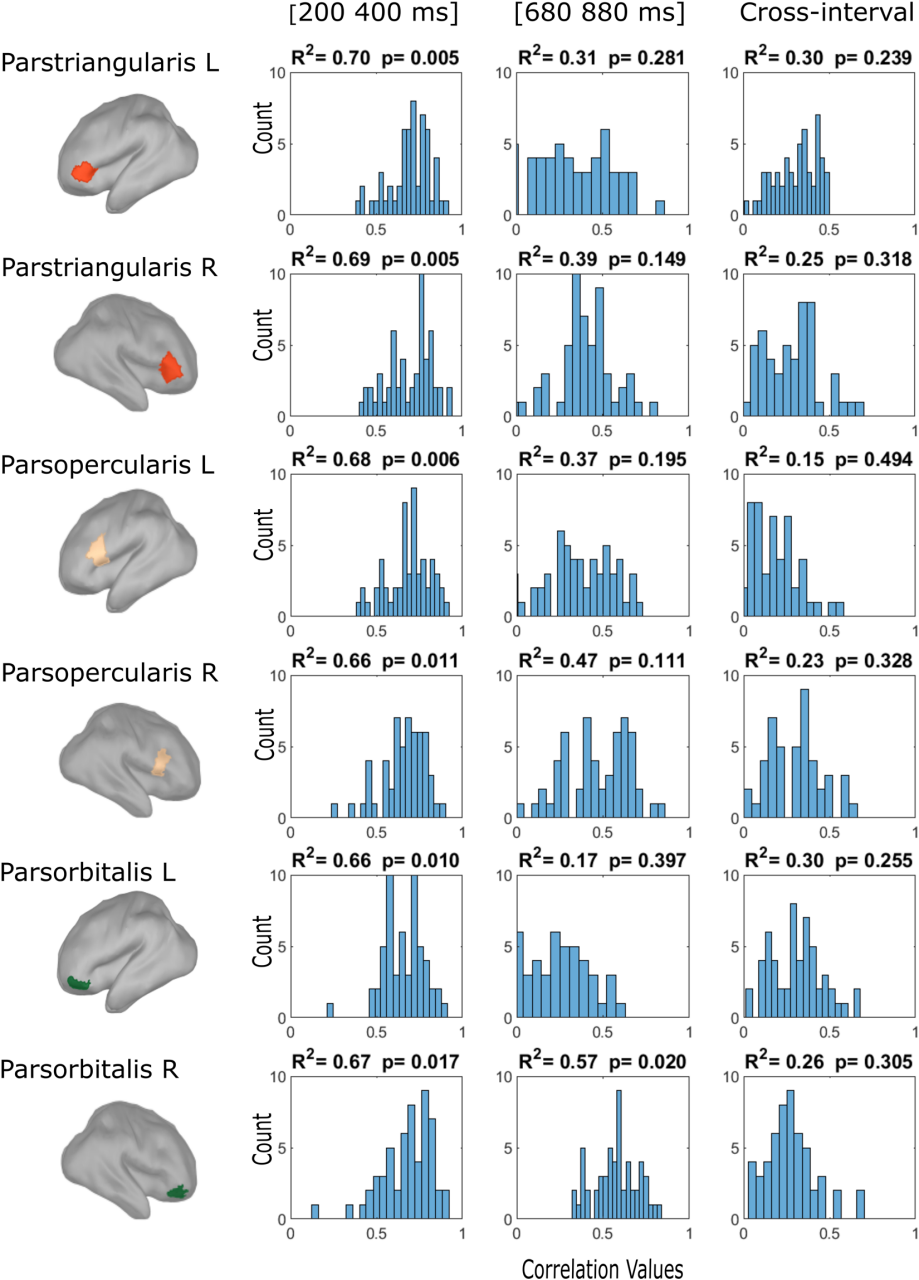
Correlation range of the differential source activity between conditions in ROIs of the IFG bilaterally. Three different correlation ranges per ROI are plotted: The left-hand graphs plot the correlation range of the differential source activity between possessive and personal pronouns within the significant pronoun interval (200–400 ms after pronoun onset), the middle graphs plot the correlation range of the differential source activity between nouns and verbs within the significant noun/verb interval (680–880 ms after pronoun onset), and the right-hand graphs plot the correlation range of the differential source activity between both intervals (i.e., the differential source activity between possessive and personal pronouns in the 200–400 ms interval correlated with the differential source activity between nouns and verbs in the 680–880 ms interval). The X-axis represents the correlation value (ranging from 0 to 1) and the Y-axis represents the amount of observations for a given correlation value (i.e., count). For all correlation graphs R-squared and p-value are given (of the averaged correlation).

In order to add support to the above argument, we measured correlations at the participant level for the significant pronoun and noun-verb time-windows in the (L)IFG to assess whether the two effects are indeed different (uncorrelated) from one another. To do so, we adopted an exhaustive correlation procedure where (1) we took two random time-points in the significant pronoun time-window (200–400 ms after pronoun-onset), calculated their difference scores (between the possessive and personal pronoun condition), correlated those scores for each individual and repeated this procedure for 56 times to obtain the correlation-range of the time-window (given that there are 8 observations in the significant time-window, this mounts to 8 times 7 correlation measures to obtain the range); then (2) we did the same procedure for the significant noun-verb interval in the (L)IFG (680–880 ms after pronoun-onset); and finally, (3) the same procedure was applied across the two significant time-window (i.e., correlation between difference score at a random time-point in the pronoun time-window and at a random time-point in the noun-verb time-window, repeated 56 times in each individual). The results of all three correlation measurements are presented in Fig. 3. The correlation range was noticeably higher within the pronoun (left panels) than in the noun-verb time-windows (middle panels), and then in the cross-interval correlating the pronoun and noun-verb effect in the (L)IFG (right panels). If the significant effect between the verbs and nouns in the (L)IFG would merely reflect the continued response to the prior pronoun effect, then one (a) would have predicted that their correlation range would overlap and not substantially drop as observed here, and (b) that the pronoun and noun-verb effects correlate. Neither was the case. These correlational observations fit the notion that the noun-verb effect in the (L)IFG unlikely reflects ‘only’ the continued response to the pronouns, but rather displays sensitivity to noun and verb activation in light of the prior pronoun response.

## Discussion

We investigated the spatiotemporal dynamics underpinning syntactic operations under predictive two-word contexts. MRI-constrained MEG sources revealed the differential activities elicited by nouns and verbs when their syntactic category could or could not be predicted based on the prior presentation of correctly corresponding pronouns (possessive for nouns and personal for verbs). We observed very early source activation differences (~80 ms) between nouns and verbs in LIFG and (apparently less strongly) in RIFG. These differences were only present when the words were preceded by their corresponding pronouns (i.e., when syntactic unification was possible). When the preceding context was non-predictive, the noun-verb contrast did not display any significant differences in IFG. In addition, the cortical activity elicited by the pronouns themselves, compared to meaningless symbols, showed similar activation differences in the left and right IFG, as well as bilateral effects in PFC. Finally, our results showed no evidence of an anterior-posterior dissociation in neural processing between verbs and nouns.

Starting with this last observation, the absence of any anterior-posterior dissociation between the verbs and the nouns was unexpected, given that we had set-up the study to utilize this contrast as a marker of grammatical class processing (see Introduction). Instead, the specific pattern observed in the (L)IFG, where nouns elicited stronger responses than verbs, replicates studies on syntactic processing where nouns and verbs were presented in inflected form within minimal grammatical contexts^33,43^. Note also, that lexical-level differences between nouns and verbs typically elicit the reverse pattern in inferior frontal regions^30^. Based on these observations, we believe our data turned out to be only sensitive to phrase-level syntactic structure building (i.e., unification) and not the lexico-semantic and morphosyntactic knowledge (i.e., memory representations) over which the unification process operates. A recent MEG study, which also included pronouns and a noun-verb contrast, did observe enhanced source activation for verbs in anterior brain regions and for nouns in posterior regions^35^. Design and stimuli differences between that and our study may account for the discrepancy in results. First, the study of Tsigka *et al*. was designed to contrast semantic and morpho-syntactic accounts of noun-verb processing differences by utilizing homonym stimuli, while our stimuli did not include homonyms. Nevertheless, it seems unlikely that this factor alone could explain the absence of noun-verb dissociations here^30^. Another difference between Tsigka *et al*.’s study and ours seems more relevant, namely the manipulation of syntactic predictability; that is, a contrast between a predictable syntactic unification context (where correct pronouns are indicative of upcoming noun or verb categories) and a context lacking such predictable syntactic structure (i.e., meaningless symbols). This factor may have caused our paradigm to be particularly sensitive to syntactic structure processing rather than lexical processing. The fact that the modulations between nouns and verbs were restricted to the (L)IFG and only occurred in the presence of prior pronoun information (i.e., when unification is possible), is indeed consistent with an explanation in terms of syntactic unification^21,28,37,40^.

Thus, while our data remain silent about the potential role of syntactic predictions on lexical activation, interestingly, they do suggest that syntactic unification processes in the (L)IFG emerge as early as 80 ms after noun/verb onset. This timing is several hundred milliseconds earlier than what is typically reported for syntactic unification^36,44–46^. Indeed, while effects linked to sentence-level analyses are typically reported to start around 250–300 ms onwards, here we see that in the presence of pronoun information, and only then, differential activation in the (L)IFG reflecting syntactic unification can already start within 100 ms of processing. One possible explanation for such remarkably early time-course is that the noun/verb effect in the (L)IFG reflect top-down signals rather than the on-line bottom up processing leading to the activation of the noun- or verb-phrase structure^28,47^.

Under this tentative explanation, the pronoun information would be used as a cue to pre-activate an upcoming syntactic structure, thereby facilitating syntactic unification operations in the (L)IFG. The processing of a pronoun (e.g., *he*/*his*) activates a syntactic context (*verb-phrase* vs. *noun-phrase*) maintained over time in the LIFG. When, subsequently, the target verb or noun is presented, it is assigned rapidly to the correct ‘slot’ thanks to the pre-activated structure, resulting in an efficient unification between prior syntactic context and current syntactic bottom-up input that is mediated the LIFG activity. Interestingly, such explanation of our results fits well with the observed direction of the effect, where nouns elicit a stronger neuromagnetic response than verbs. That is, the effect’s direction can be explained along similar lines as has been proposed for the lexico-semantic N400 priming effect, where higher lexico-semantic overlap between context and target result in smaller electrophysiological amplitude^48,49^. Adapted to the minimal grammatical context used in the current study, the presentation of a possessive pronoun can only predict an upcoming noun, while the presentation of a personal pronoun predicts an upcoming verb as well as its inflection (for person and number; e.g., in English: *you RUN* vs. *he RUN***S**; *with richer inflections in French, the language used here*). In this sense, the ‘predictive value’ of a personal pronoun would be higher (more specific) than of a possessive pronoun. Consequently, there is more neuronal overlap between the syntactic context of the personal pronouns and the syntactic information of the bottom-up verb input, generating a reduced amplitude compared to the presentation of the noun input in the presence of a possessive pronoun context.

That said, we haste to add and stress again that this account is only a post-hoc tentative (though interesting) explanation of our data; the current study does not prove this to be correct. Indeed, our crucial manipulation to inform on predictive processing concerned the anterior-posterior dissociation between nouns and verbs (where we predicted that earlier noun-verb cortical dissociations would index linguistic prediction), but, as discussed above, we did not observe such anterior-posterior dissociations in response to the noun and verb stimuli. Hence, the sole data-point allowing us to suggest that the rapid unification effects we observed in the (L)IFG could be due to predictive top-down processing concerns the speed (within 100 ms after noun/verb presentation when syntactic unification is possible). While we believe this data-point is certainly suggestive and finds a more parsimonious explanation under a predictive unification account, future research is necessary which explores and tests this possibility as an a-priori hypothesis. For now, the main observation and conclusion of our study is that grammatical class modulates the (L)IFG within 100 ms of processing when the syntactic context is predictive.

This main observation of our study is interesting, because it constrains neurolinguistic theories of syntactic parsing. In particular, a mechanism should be in place that can explain why grammatical class modulates the brain activity in the (L)IFG so rapidly when (predictive) syntactic unification is possible. In this regard, it is notable that we observe the interaction effect between predictability and grammatical category solely in inferior frontal brain regions, and not in other syntax sensitive regions in the temporal cortex such as the anterior temporal lobe (ATL), where recent work shows an involvement for computing basic aspects of sentence-level syntax in language comprehension^41,50–52^. While the current null-results for the noun-verb contrast in the temporal cortex do not negate a potential role of the ATL in basic combinatorial syntax, the fact that the simple combination of pronoun and noun/verb information modulates activity in the (L)IFG *does* indicate that also inferior frontal brain regions are recruited for basic sentence-level computations, as argued by the memory and unification account of Broca’s region^21,28^.

Thus far we have interpreted this effect of early noun/verb differences in the (L)IFG when preceded by pronoun information in function of the second set of predictions as specified in the Introduction, namely syntactic unification. However, one may wonder whether this effect reflects semantic unification of verb- versus noun-phrases rather than syntactic unification. Though we did not manipulate the semantic content between these conditions explicitly, it is not unconceivable that there are systematic semantic differences between the noun- and verb-phrases (e.g., action-relatedness). If so, our data would index that the integration of nouns and verbs in phrasal contexts occurs within 100 ms in the (L)IFG. While an interesting alternative explanation (but note that the manner to explain ‘why’ such semantic integration would occur that early in the (L)IFG, would be qualitatively similar to the one given for syntactic unification above), we believe that accounting for the data in terms of syntactic unification is the more parsimonious option. This is because under a semantic account (a) one should expect to (also) see differences in well-known temporal brain regions sensitive to semantic integration^48,53,54^, and (b) one would expect the reversed pattern in the (L)IFG with verbs eliciting enhanced source activation compared to nouns^30^. Instead, the pattern found here is consistent with other studies on syntactic processing^33,43^. Based on these data, we believe our results are best understood as showing rapid syntactic unification in the (L)IFG.

A final interesting result from our study concerns the increased activation for the possessive pronouns (i.e. preceding nouns) versus personal pronouns (i.e. preceding verbs) between 200 and 400 ms after their onset, in the left and right IFG as well as in the superior frontal (SFC), anterior cingulate and orbitofrontal cortices (OFC) bilaterally. These effects are interesting given that pronoun representations are typically associated with left perisylvian language areas^36,55^, and not with the PFC. Instead, regions in the PFC are known to serve domain-general functions, often associated with executive functioning, and, for regions such as SFC and OFC, particularly with selective attention, expectation, planning, and proactive top-down processing^56–59^. Considering the current design and the rapid speed with which nouns and verbs activated the (L)IFG, these pronoun-driven modulations in SFC and OFC may suggest the involvement of a domain-general top-down network in the proactive processing of language^60^. Such interpretation would be consistent with recent fMRI findings showing prefrontal activations in the predictive processing of sentence and story comprehension^61,62^ (see also for such domain-general top-down processing of language in parietal and subcortical regions)^63,64^. Furthermore, the fine-grained temporal information available in the current MEG experiment revealed that the prefrontal activations elicited by the pronouns were detected in the same time windows than the pronoun modulations in (L)IFG. Based on this finding, rapid syntactic unification processes in the (L)IFG may be aided by top-down modulations stemming from domain-general processing regions in the PFC, where the (L)IFG dynamically binds with regions in the prefrontal cortex capable of biasing the neuronal activity of the system with which it is in synchrony^65–68^. Future research should establish whether the prefrontal activation patterns as observed here are indeed causally involved in the top-down processing of syntactic unification in the (L)IFG.

In sum, the current study demonstrated that syntactic processing in the (L)IFG occurs very fast, within 100 ms of stimulus presentation, at least for the minimal grammatical contexts used here. This early time-course sets an important constraint for neurobiological models of syntax: a mechanism has to be in place allowing for such very early unification of different syntactic representations. One mechanism which could potentially account for this speed, we argued, is top-down driven predictive coding. Such mechanism would nicely fit with the observation that the pronouns preceding the rapid noun-verb modulations activated simultaneously the (L)IFG and top-down control regions in the PFC. This tentative interpretation raises interesting novel questions and hypotheses to pursue in future research: Is syntactic unification in the (L)IFG driven by prediction? And is the mechanism responsible for those predictions sustained by the synchronous and dynamical binding of the (L)IFG with brain regions in the PFC capable of sending top-down biasing signals?

## Materials and Methods

The study received appropriate ethical approval (filed under id “RCB: 2011-A00562–39” at the regional ethical committee “Comité de Protection des Personnes Sud Méditerranée I”), and was conducted in accordance with the relevant guidelines and regulations. Furthermore, to conform with Open Research Practices in science, a detailed description of the project, all the materials and design specifications, the raw MEG data files, MRI images of all participants, the source code and pre-processing pipelines (BrainStorm), and the source reconstruction protocol are publically archived and freely available via the ‘SharedProjects’ repository of the BLRI/ILCB (link: https://blricrex.hypotheses.org/le-crex/sharedprojects). The Open Data and Open Materials available for our study should allow for easy and complete independent replication of our analysis and results.

### Participants

22 native speakers of French, all students at Aix-Marseille University (mean age = 22.4 years, SD = 4.0) took part in the experiment. Before the experimental session, the participants were given details about the procedure and they provided their written informed consent. All participants were right-handed, did not suffer from neurological disorders, and had normal or corrected-to-normal vision. They received monetary compensation for their participation in the experiment.

### Stimuli

The target stimuli consisted of French words drawn from the Lexique database (v3.80)^69^. They were either function words (possessive and nominative personal pronouns) or content words (nouns and verbs). The pronouns used in the experiment are detailed in Table 1. They were selected to include and balance grammatical gender, number and person across the noun and verb conditions.

**Table 1:** Matched possessive and personal pronouns used as stimuli.

Possessive (before nouns)				Personal (before verbs)				Number	Person
FRENCH	Translation	Gender	Length	FRENCH	Translation	Gender	Length		
MA	my	fem	2	JE	I	neut	2	Sing	1^st^
TA	your	fem	2	TU	you	neut	2		2^nd^
SON	his	masc	3	IL	he	masc	2		3^rd^
SA	his	fem	2	ELLE	she	fem	4		3^rd^
VOTRE	your	neut	5	VOUS	you	neut	4	Plur	2^nd^
LEURS	their	neut	5	ELLES	them	fem	5		3^rd^

In the “Predictive” condition, possessive pronouns were presented before nouns while personal pronouns were presented before verbs. In the “Non-predictive” condition, the pronouns were replaced by series of hash marks (“#”) matched in length of characters.

Once the function words had been selected, nouns and verbs matching for their number, person and gender were drawn from the database^69^. The selection process was performed automatically using a pseudo-random procedure with the following constraints. Targets were filtered to have a lexical frequency of more than 10 occurrences per million, be between 3 and 8 letters in length, have no homographs (i.e. multiple readings, as “to/the watch”), not have a vowel following a vowel in the closed-class word (which would have been inappropriate in French, where *“je aime” contracts to “j’aime”). Any profane words were manually excluded at this stage. Selected nouns and verbs were first pooled by length, and then pair-matched for lexical frequency using Euclidean distance matching to find the optimal set of matches. Pairs were excluded if the orthographic distance between the members (OLD20)^70^ or average bigram frequency exceeded 2 S.D. of the variance in the length pool, or if the difference in their number of phonemes was greater than 1. No open-class words were duplicated. These pairs were checked for validity by a group of 6 native French speakers to exclude unnatural seeming stimuli, or those with unusual connotations or valence. From the list of stimuli that survived this filtration, the 370 noun/verb pairs (740 words) best matching each other for lexical frequency were chosen as a final list.

In addition, twenty ‘catch’ pseudo-word trials were included per condition (i.e., 11% of the trials), consisting of a closed class word or a #-string, followed by a pseudo-word. The pseudo-words had been generated from the Lexique toolbox using 40 words (20 nouns, 20 verbs) chosen from the open-class stimuli. Such words were chosen randomly but in proportion to the frequency of each function word in the stimulus set. During the experiment, participants were instructed to detect the presentation of pseudo-word items.

### Design

The experiment had a 2 × 2 design, where the factor Syntactic Predictability (“Predictive” versus “Non-predictive” contexts) was crossed with the factor Grammatical Category (“Nouns” versus “Verbs”). In the “Predictive” condition, a trial consisted of a function word followed by an appropriate content word, either a noun or a verb (e.g. TA CHAISE / TU PRENDS; “your chair”, “you take”). In the “non-predictive” condition, a row of hash marks ([####]) was followed by a content word, either a noun or a verb (e.g. ## CHAISE/## PRENDS; “## chair”, “## take”). The number of #-strings of different lengths in the non-predictive condition was balanced against the number of closed-class words with a given length in the predictive condition. There were 185 trials per condition; each of the 370 nouns and 370 verbs appeared only once per participant.

The experimental lists were constructed as follows. Starting from the original stimulus pool of 370 noun/verb pairs, various pairs of lexical frequency matched lists were created using the software “Match”^71^. The lists were counterbalanced across conditions between participants, so that all nouns and verbs equally appeared in the Predictive and Non-predictive conditions. To generate actual trial orders for each participant, the lists were sorted into pseudo-random orders with the following constraints: (1) No more than 4 occurrences of each grammatical category (noun or verb) followed in sequence; (2) no more than 4 occurrences of each predictive condition (“Predictive” or “Non-predictive”) followed in sequence; (3) the same function word never appeared twice in a row; (4) there were at least 5 experimental trials between catch trials. In this manner, a randomized list of 780 trials was created for each participant. Of those 780 trials, 80 corresponded to the pseudo-word catch trials (40 with pronouns as primes and 40 with symbols as primes), 370 corresponded to the Predicted Noun and Verb design cells (with the corresponding possessive or personal pronoun prime preceding the noun or verb target), and 370 corresponded to the Unpredicted Noun and Verb design cells (with meaningless symbol primes preceding the noun or verb target).

### Procedure

The visual materials were displayed using Neurobehavioural Systems’ ‘Presentation’ software. Participants were given written instruction, as well as oral clarifications as needed, that they should read the two stimuli presented in sequence. They were asked to press a button on a response box with their right index finger in any trial where the second stimuli they saw was a pseudo-word. A button press at any time other than in the 1,000 ms period after pseudo-word presentation would immediately produce negative feedback, would cause the protocol to skip to the next section of the trial, and the trial to be excluded from the analysis. All experimental stimuli were presented in black Arial font at size 48 on a 50% grey background, words centered on screen between two static lines. Experimental trials proceeded as follows: (1) A pair of nonius vertical lines appeared alone for a randomly (flat distribution) jittered amount of time between 1,000 and 2,000 milliseconds; (2) a fixation cross appeared between the lines for 500 ms; (3) a function word or #-string appeared for 600 ms; (4) a content word appeared for 600 ms. The catch trials proceeded equally except that their final stimulus was a pseudo-word, upon which a button press was required. These catch trials were included to ensure that participants attentively processed the stimuli in the experiment and to ensure that they engaged in a task (i.e., lexical decision) which is orthogonal to the contrast of interest (i.e., nouns versus verbs). In this manner, the goal-directed behavior of the participants is geared towards the identification of pseudo-words, excluding any strategic advantages tied to task performance for predicting the syntactic category of the word-stimuli. Analyses of the data (see also below) only involved the nouns and verbs (i.e., no-go trials); pseudo-words (i.e., go-trials) were thus excluded from data analyses. If a button press was made within 1,000 ms of the pseudo-word’s appearance, positive feedback (a green tick-mark between the nonius lines) was shown for 400 ms, otherwise negative feedback (a red cross between the nonius lines) was shown for 400 ms.

### MEG recordings

While participants performed the task, continuous MEG of spontaneous cerebral activity was recorded using a 4D Neuroimaging Magnes 3600 Whole Head 248 Channel scanner (Timone Hospital, Marseille, France). Data were sampled at 2034.15 Hz. Head shape and position coil location were recorded using a Polhemus Fastrak 3-D digitizing stylus at the beginning of the recording session. Head position was measured at the beginning and the end of each run (6 runs per subject). We ensured that the position of the sensor regarding the subject did not change during the run and between the runs more than 3 mm. Electro-oculogram (EOG) and electrocardiogram (ECG) were recorded simultaneously for the offline rejection of eye movements and cardiac artifacts. The distance between the participant’s eyes and the screen on which stimuli were displayed was 81 cm (mean measurement, SD 2 cm). A trigger square invisible to the participant was projected onto a photodiode which was used to signal the presence of a function word stimulus on-screen and to synchronize the MEG and EOG/ECG recordings.

### MRI pre-processing

The cortical surface was reconstructed for each participant using FreeSurfer software (http://freesurfer.net) from their high-resolution 3D T1-weighted MRI structural image (3 T Brucker, Timone Hospital, Marseille, France).

### Anatomical ROI analyses

The Brainstorm software^72^ used for MEG analysis includes a subdivision of individual anatomy into regions of interest based on the anatomical atlases of Desikan *et al*.^73^. All 68 cortical scouts of this atlas, and 4 sub-cortical scouts (left and right hippocampi and amygdalae) were selected for the analyses. The temporal regions (superior, middle and inferior) are very elongated in this atlas. In keeping with the granularity of recent descriptions of the language network^74^, they were subdivided post-hoc into anterior, mid, and posterior sections. The cortex surface was defined with 15000 vertices and the MRI was realigned on three fiducials (nasion, left and right pre-auricular point) and then refined with the superposition of the segmentation of the scalp from the MRI and the head shape acquired prior to the recording.

### MEG data processing

The MEG data were first filtered by a band-pass filter in the range of 0.3–300 Hz (Butteworth IIR filter, 2-order filter and zero-phase forward and reverse filter) using Anywave software^75^. Then, functional and structural data were exported into Brainstorm to perform preprocessing, and the analysis of cortical sources and anatomical regions of interest (ROI). The rejection of system artifacts on the MEG signal were performed visually on the basis of the combination of the power spectrum density (Welch) and the 3D sensor topography. The cardiac and ocular artifacts were detected and removed using signal space projection (SSP). The signal was filtered by a low-pass filter of 40 Hz and corrected with a baseline defined between −200 ms and 0 milliseconds. Event-Related Fields (ERFs) were time-locked to the onset of the first word of each trial on the screen. The epochs were segmented between −200 and 1200 ms around that time. Outlier trials were first automatically detected using a maximum amplitude cut-off of 3000 fT (‘process_detectbad’ in Brainstorm) to identify potentially bad trials. Then, an additional manual inspection was performed on each trial to classify it as good or bad. Only trials that were artefact-free and that had not generated a false alarm (i.e. a behavioral error) were included in the sensor averaging.

The sensor averaging was computed for each participant and represented the mean time courses per channel over the epochs of each experimental condition (Predicted Nouns, Predicted Verbs, Unpredicted Nouns and Unpredicted Verbs). The cortical sources of the corresponding neuromagnetic activity were calculated using Minimum-Norm Current Estimates (wMNE) with constraints on source orientations (normal to the cortex). The forward modeling method was overlapping spheres, the number of vertices (i.e. dipoles) used for source estimation was about 15,000. For each dipole, the time course was normalized relative to the baseline (−200 to 0 ms) to compute an absolute z-score. Finally, the source time-series (at vertices) were summarized by averaging within each anatomical ROI for each experimental design cell. This yielded four time-series (Predicted nouns, Predicted Verbs, Unpredicted nouns, and Unpredicted Verbs) per anatomical ROI per participant.

### Statistical analyses

ROI time series were imported in Matlab for statistical analyses and reporting. Before computing statistics, the ROI time series were down sampled to 40 Hz using the function ‘resample’ of Matlab software (i.e., to analyze the data in averaged time bins of 25 ms from stimulus onset over the entire epoch; 0–1200 ms). We conducted a mass univariate analyses on the source time-series of every ROI (i.e., a whole-head analyses; for more details on mass univariate analyses see)^76^, for consecutive time points of 25 ms (i.e., minimal time-window for analyses given the 40 Hz low-pass filter)^77^. The approach thus consists of many non-parametrical statistical t-tests (based on 1000 permutations) performed with the EEGLAB toolbox^78^ and aims to compare the experimental conditions at group-level for a given ROI. The measure of comparison is one timepoint across subjects (22 subjects) per ROI and per condition. The resultant p-value is corrected for multiple comparisons (False Discovery Rate)^79^.
